# Assessing Markers of Reproducibility and Transparency in Smoking Behaviour Change Intervention Evaluations

**DOI:** 10.1155/2021/6694386

**Published:** 2021-01-15

**Authors:** Emma Norris, Yiwei He, Rachel Loh, Robert West, Susan Michie

**Affiliations:** ^1^Health Behaviour Change Research Group, Department of Health Sciences, Brunel University, UK; ^2^Centre for Behaviour Change, University College London, UK; ^3^Psychology & Language Sciences, University College London, UK; ^4^Research Department of Epidemiology & Public Health, University College London, UK

## Abstract

**Introduction:**

Activities promoting research reproducibility and transparency are crucial for generating trustworthy evidence. Evaluation of smoking interventions is one area where vested interests may motivate reduced reproducibility and transparency.

**Aims:**

Assess markers of transparency and reproducibility in smoking behaviour change intervention evaluation reports.

**Methods:**

One hundred evaluation reports of smoking behaviour change intervention randomised controlled trials published in 2018-2019 were identified. Reproducibility markers of pre-registration; protocol sharing; data, material, and analysis script sharing; replication of a previous study; and open access publication were coded in identified reports. Transparency markers of funding and conflict of interest declarations were also coded. Coding was performed by two researchers, with inter-rater reliability calculated using Krippendorff's alpha.

**Results:**

Seventy-one percent of reports were open access, and 73% were pre-registered. However, there are only 13% provided accessible materials, 7% accessible data, and 1% accessible analysis scripts. No reports were replication studies. Ninety-four percent of reports provided a funding source statement, and eighty-eight percent of reports provided a conflict of interest statement.

**Conclusions:**

Open data, materials, analysis, and replications are rare in smoking behaviour change interventions, whereas funding source and conflict of interest declarations are common. Future smoking research should be more reproducible to enable knowledge accumulation. This study was pre-registered: https://osf.io/yqj5p.

## 1. Introduction

Researchers are becoming increasingly aware of the importance of reproducibility and transparency in scientific research and reporting [1, 2]. A well-documented “replication crisis” in psychology and other disciplines has shown that engrained academic incentives encouraging novel research have led to biased and irreproducible findings [3–6]. Researchers, journals, and funding organisations across psychology and health sciences are contributing to reforming scientific practice to improve the credibility and accessibility of research [1, 7].

“Open Science,” where some or all parts of the research process are made publicly and freely available, is essential for increasing research transparency, credibility, reproducibility, and accessibility [8]. Reproducibility-facilitating research behaviours are varied and occur throughout the research life cycle. During study design, pre-registration and protocols specify the hypotheses, methods, and analysis plan to be used in proposed subsequent research in repositories such as Open Science Framework and AsPredicted. Such specification is designed to reduce researcher degrees of freedom and undisclosed flexibility, ensuring features such as primary and secondary hypotheses and analysis plans remain fixed and preventing “p-hacking” [9]. Within health research, pre-registration and protocol sharing also facilitate future replication and real-world adoption of medical and behavioural interventions [10]. During data analysis, scripts can be made more reproducible by marking their code with step-by-step comments, improving clarity and replication [11]. During dissemination, materials (such as intervention protocols and questionnaires), data, and analysis scripts can be made available by uploading to repositories such as Open Science Framework or GitHub [12], facilitating the replication of effective research and interventions [13]. Allowing data and trial reports to be made available regardless of their findings enables a more accurate picture of the full state of research, minimising the “file drawer” problem by which positive findings are more likely to be published than negative findings [14]. Sharing data and analysis code also allows for checking of research findings and conclusions, as well as easier synthesis of related findings via meta-analyses [15]. Transparency-facilitating research behaviours include reporting sources of research funding and conflicts of interest [16, 17]. These are important in that they help readers to make informed judgements about potential risks of bias [18].

Metascience studies have assessed markers of reproducibility and transparency in the related domains of psychology and life sciences. A recent study exploring 250 psychology studies of varying study designs published between 2014 and 2017 found transparency and reproducibility behaviours to be infrequent [19]. Although public availability of studies via open access was common (65%), sharing of research resources was low for materials (14%), raw data (2%), and analysis scripts (1%). Pre-registration (3%) and study protocols (0%) were also infrequent [19]. Transparency of reporting was inconsistent for funding statements (62%) and conflict of interest disclosure statements (39%) [19]. Metascience studies have assessed reproducibility and transparency across other disciplines, including 250 studies in social sciences [20], 149 studies in biomedicine [21], and 480 studies across two journals in biostatistics [22], all with no restrictions on study designs. Other research has focused on the prevalence of specific reproducibility behaviours, such as the prevalence of open access publications, finding about 45% across scientific discipline assessed in 2015 [23].

However, the extent of reproducibility and transparency behaviours in public health research, including smoking cessation, is currently unclear. A recent investigation of randomised controlled trials addressing addiction found data sharing to be nonexistent. 0/394 trials were found to make their data publicly available, with 31.7% of included trials addressing tobacco addiction [24]. It must be noted that various persistent barriers to data sharing exist, including technical, motivational, economic, political, legal, and ethical considerations (van Panhuis et al., 2014), which may limit the uptake of this specific Open Science behaviour. Markers of wider reproducibility behaviours are yet to be assessed in addiction research.

Transparent reporting in terms of funding and conflicts of interest is especially crucial for smoking cessation, where tobacco and pharmaceutical companies fund some research directly or indirectly [25]. Such vested interests may distort the reporting and interpreting of results, and this may especially be the case in areas of controversy such as e-cigarette research [13, 17, 26, 27]. The aim of the current study is to assess markers of (i) reproducibility and (ii) transparency within smoking intervention evaluation reports.

## 2. Methods

### 2.1. Study Design

This was a retrospective observational study with a cross-sectional design. Sampling units were individual behaviour change intervention reports. This study applied a methodology used to assess reproducibility and transparency in the wider psychological sciences [19] and social sciences [20] to the context of smoking randomised controlled trial intervention reports. This study was pre-registered: https://osf.io/yqj5p. All deviations from this protocol are explicitly acknowledged in the appendix.

### 2.2. Sample of Reports

The Cochrane Tobacco Group Specialised Register of controlled trials was searched in November 2019, identifying 1630 reports from 2018 to 2019. Inclusion criteria were randomised controlled trials published in 2018 and 2019. Exclusion criteria were trial protocols, abstract-only entries, and economic or process evaluations. Of the 157 reports remaining after applying these criteria, 100 reports were selected due to time and resource constraints using a random number generator. PDFs were obtained from journal websites. These reports were also already included in the ongoing Human Behaviour-Change Project ([28, 29], https://osf.io/efp4x/), working to synthesis published evidence in behaviour change, beginning with smoking intervention evaluations. A list of all 100 reports included in this study is available: https://osf.io/4pfxm/.

### 2.3. Measures

Article characteristics extracted in this study were as follows: (i) 2018 journal impact factor for each report using the Thomson Reuters Journal Citation Reports facility and (ii) country of the corresponding author (Table 1). Additional article characteristics already extracted as part of the Human Behaviour-Change Project are also reported: (iii) smoking outcome behaviour (smoking abstinence, onset, reduction, quit attempt, or second-hand smoking) and (iv) behaviour change techniques (BCTs) in the most complex intervention group, coded using the Behaviour Change Techniques Taxonomy v1 [30]. In short, data from the Human Behaviour-Change Project was extracted using EPPI-Reviewer software [31] by two independent reviewers before their coding was reconciled and agreed. The full process of manual data extraction within the Human Behaviour-Change Project [32]. All extracted data on included papers is available: https://osf.io/zafyg/.

Markers of research reproducibility were assessed by recording the presence of the following in included reports: (i) *pre-registration*: whether pre-registration was reported as carried out, where the pre-registration was hosted (e.g., Open Science Framework and AsPredicted), whether it could be accessed, and what aspects of the study were pre-registered; (ii) *protocol sharing*: whether a protocol was reported as carried out and what aspects of the study were included in the protocol; (iii) *data sharing*: whether data was available, where it was available (e.g., online repository such as Open Science Framework, upon request from authors, as a journal supplementary file), whether the data was downloadable and accessible, whether data files were clearly documented, and whether data files were sufficient to allow replication of reported findings; (iv) *material sharing*: whether study materials were available, where they were available (e.g., online repository such as Open Science Framework, upon request from authors, as a journal supplementary file), and whether the materials were downloadable and accessible; (v) *analysis script sharing:* whether analysis scripts were available, where they were available (e.g., online repository such as Open Science Framework, upon request from authors, as a journal supplementary file), and whether the analysis scripts were downloadable and accessible; (vi) *replication of a previous study*: whether the study claimed to be a replication attempt of a previous study; and (vii) *open access publication:* whether the study was published as open access.

Markers of research transparency were assessed by recording the presence of the following in included reports: (i) *funding sources*: whether funding sources were declared and if research was funded by public organisations (such as research councils or charities), pharmaceutical, tobacco, or other companies; (ii) *conflicts of interest*: whether conflicts of interest were declared and whether conflicts were with public organisations (such as research councils or charities), pharmaceutical, tobacco, or other companies. All measured variables are shown in Table 1.

### 2.4. Procedure

Data collection took place between February and March 2020. Data for all measures were extracted onto a Google Form (https://osf.io/xvwjz/). All reports were independently coded by two researchers. Any discrepancies were resolved through discussion, with input from a third researcher if required.

### 2.5. Analysis

Research reproducibility was assessed using the markers of pre-registration; sharing of protocols, data, materials, and analysis scripts; replication; and open access publishing (Table 1). Research transparency was assessed using the markers of funding source and conflicts of interest declarations. Inter-rater reliability of the independent coding of the two researchers was calculated using Krippendorff's alpha [33] using Python 3.6 (https://github.com/HumanBehaviourChangeProject/Automation-InterRater-Reliability).

## 3. Results

Inter-rater reliability was assessed as excellent across all coding, *a* = 0.87. Full data provided on OSF: https://osf.io/sw63b/.

### 3.1. Sample Characteristics

Seventy-one out of 100 smoking behaviour change intervention reports were published in 2018 and 29 published in 2019. Out of the 100 reports, four had no 2018 journal impact factor, with the remaining 96 reports having impact factors ranging from 0.888 to 70.67 (mean = 4.95). Fifty-four out of 100 reports took place in the United States of America (https://osf.io/j2zp3/). Data from the Human Behaviour-Change Project identified that out of the 100 reports, 94 had a primary outcome behaviour of smoking abstinence, two of smoking onset and smoking reduction, respectively, and one of quit attempts and second-hand smoking, respectively. Forty-six out of the total 93 behaviour change techniques (BCTs) within the Behaviour Change Techniques Taxonomy (BCTTv1) were identified in the included reports. An average of 4.41 BCTs was identified in each report. The most commonly identified BCTs were as follows: social support (unspecified) (BCT 3.1, *n* = 65/100), pharmacological support (BCT 11.1, *n* = 61/100), problem solving (BCT 1.2, *n* = 42/100), and goal setting (behaviour) (BCT 1.1, *n* = 34/100). A figure of all outcome behaviour and BCT codings can be found: https://osf.io/6w3f4/.

### 3.2. Markers of Reproducibility in Smoking Behaviour Change Intervention Evaluation Reports

Final reconciled coding of reproducibility and transparency for all smoking behaviour change intervention reports can be found at https://osf.io/jcgx6/.

#### 3.2.1. Article Availability (Open Access)

**Figure 1:**
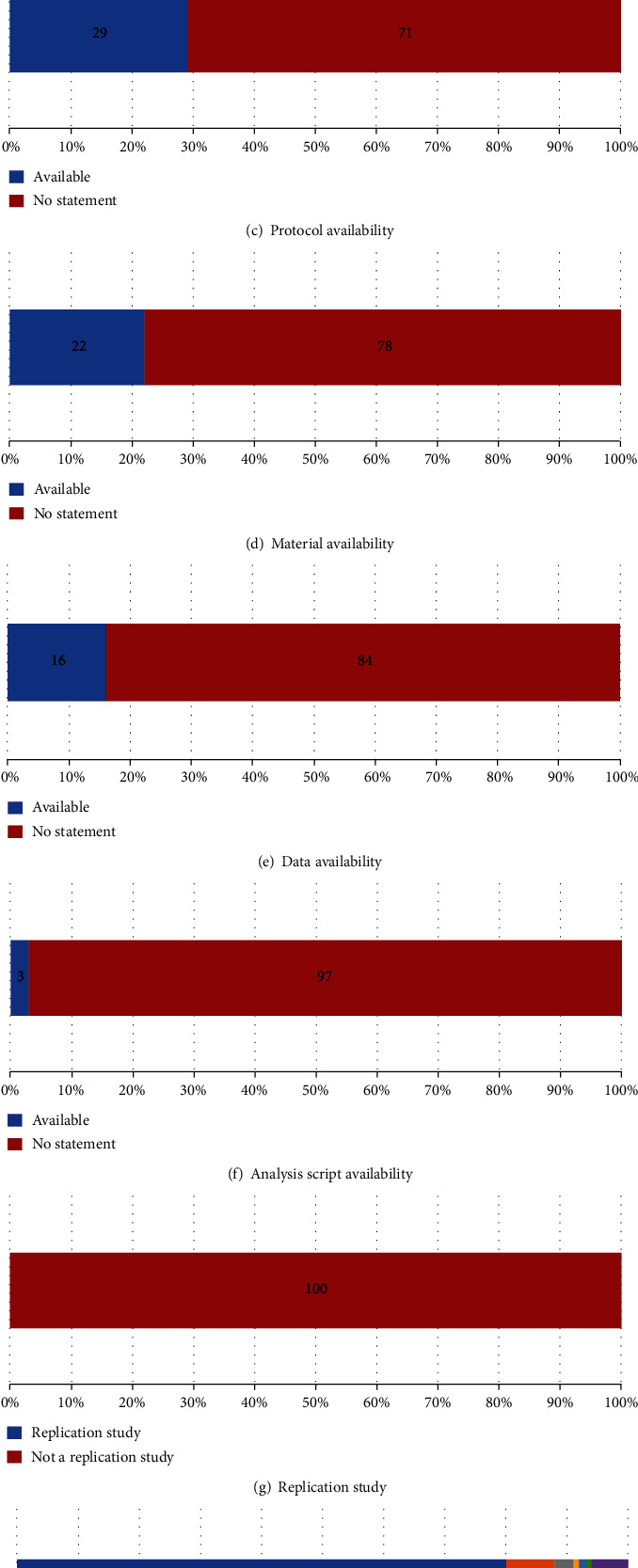


Seventy-one out of 100 smoking behaviour change intervention reports were available via open access, with 29 only accessible through a paywall (Figure 1(a)).

#### 3.2.2. Pre-registration

Seventy-three out of 100 smoking behaviour change intervention reports stated that they were pre-registered, with 72 of these being accessible. Fifty-four studies were pre-registered at ClinicalTrials.gov, with the remainder pre-registered at the International Standard Randomized Clinical Trial Number registry (ISRCTN; *n* = 7), the Australian and New Zealand Clinical Trials Registry (ANZCTR; *n* = 4), Chinese Clinical Trial Registry (ChCTR; *n* = 2), Netherlands Trial Register (NTR; *n* = 2), Iranian Clinical Trials Registry (IRCT; *n* = 1), Clinical Research Information Service in Korea (CRIS; *n* = 1), or the UMIN Clinical Trials Registry in Japan (UMIN-CTR; *n* = 1).

All of the 72 accessible pre-registrations reported methods, with 2 also reporting hypothesis. Only two accessible pre-registrations included hypothesis, methods, and analysis plans. Twenty-six of the 100 reports did not include any statement of pre-registration. One report stated the study was not pre-registered (Figure 1(b)).

#### 3.2.3. Protocol Availability

Seventy-one out of 100 smoking behaviour change intervention reports did not include a statement about protocol availability. For the 29 reports that included accessible protocols, 23 had a protocol that included hypothesis, methods, and analysis plans. Three reports only had methods in their protocol, whereas two of them included both hypothesis and methods, and one of them included methods and analysis plans (Figure 1(c)).

#### 3.2.4. Material Availability

Twenty-two out of 100 reports included a statement saying the intervention materials used were available. Sixteen of these reports provided materials via journal supplementary files, and six reports stated that their materials were only available upon request from the authors (Figure 1(d)).

#### 3.2.5. Data Availability

Sixteen out of 100 reports included a data availability statement. Nine reports stated data was available upon request from the authors, and one stated the data was not available. The remaining six articles included their data in the supplementary files hosted by the journals, but one article's data file could not be opened. Four of the remaining articles had clearly documented data files, but only two of them contained all necessary raw data. As such in total, only seven reports provided links to data that was actually accessible (Figure 1(e)).

#### 3.2.6. Analysis Script Availability

Three out of 100 reports included an analysis script availability statement. However, only one provided accessible script as a supplementary file, with the remaining two stating analysis script available upon request from authors (Figure 1(f)).

#### 3.2.7. Replication Study

None of the 100 smoking behaviour change intervention reports were described as replication studies (Figure 1(g)).

### 3.3. Markers of Transparency in Smoking Behaviour Change Intervention Evaluation Reports

Final reconciled coding of reproducibility and transparency markers for all smoking behaviour change intervention reports can be found at https://osf.io/jcgx6/.

#### 3.3.1. Funding

Ninety-four of the 100 smoking behaviour change intervention reports included a statement about funding sources. Most of the reports disclosed public funding only such as via government-funded research grants, charities, or universities (*n* = 80). Eight reports disclosed both public funding and funding from private companies. Five reports disclosed funding from private companies only, including pharmaceutical (*n* = 3), tobacco companies (*n* = 1), and other companies (*n* = 1). One report reported receiving no funding (Figure 1(h)).

#### 3.3.2. Conflicts of Interest

Eighty-eight of the 100 articles provided a conflict of interest statement. Most of these reports reported that there were no conflicts of interest (*n* = 51). Thirty-seven reports reported that there was at least one conflict of interest, including from a pharmaceutical company (*n* = 27), private company (*n* = 17), public organisation (*n* = 13), and tobacco company (*n* = 3) (Figure 1(i)).

## 4. Discussion

This assessment of 100 smoking behaviour change intervention evaluation reports identified varying levels of research reproducibility markers. Most reports were open access and pre-registered; however, research materials, data, and analysis scripts were not frequently provided and no replication studies were identified. Markers of transparency assessed here by funding source and conflicts of interest declarations were common.

### 4.1. Assessment of Reproducibility Markers in Smoking Behaviour Change Intervention Evaluation Reports

Pre-registration, as a marker of research reproducibility, was found to be higher for smoking RCTs (73%) than in wider psychological research of varying study designs (3%) [19]. Open access reports were at similarly moderate levels (71%) to psychology (65%) [19], but greater than the 45% observed in the social sciences [20], 25% in biomedicine [21], and 45% across scientific literature published in 2015 [23]. This high rate of open access publishing in smoking interventions may reflect increasing requirements by health funding bodies for funded researchers to publish in open access outlets [34, 35] and increasing usage of preprint publication outlets such as PsyArXiv for the psychological sciences and medRxiv for medical sciences.

The proportion of open materials was lower than in biomedicine (13% vs. 33%) [21] but similar to the 11% of the social sciences [9]. Open analysis scripts were found to be as infrequently provided in smoking interventions as in wider psychological research (both 1%) [19], social sciences [20], and biostatistics [22].

Open data of smoking interventions was found to be very low (7%), but greater than the 0% estimate in a larger sample of 394 smoking RCTs [24] and to the 2% of wider psychological research [19]. Raw data are essential for meta-analyses to make sense of the diverse smoking cessation evidence. Common barriers for including studies in meta-analyses include a lack of available data, often after requests from authors [36, 37]. Provision of raw data as supplementary files to published intervention reports or via trusted third-party repositories such as the Open Science Framework [12] is important to facilitate evidence synthesis, especially in a field as important for global health as smoking cessation.

No replication attempts were identified in this sample of smoking intervention reports, compared to 5% in wider psychology studies [19] and 1% in the social sciences [20]. This lack of replication may be due to a lack of available resources of smoking interventions to facilitate replication, as identified in this study, or may reflect a lack of research prioritisation and funding for replication, with novel rather than confirmatory research prioritised at global, institutional levels [1, 6].

### 4.2. Assessment of Transparency Markers in Smoking Behaviour Change Intervention Evaluation Reports

Declaration of funding sources and conflicts of interest, as markers of research transparency, was found here to be commonly provided in smoking intervention evaluation reports. Funding sources were declared in more smoking reports (95%) than wider psychology (62%) [19], social sciences (31%) [20], and biomedical science reports (69%) [21]. Similarly, a statement on conflicts of interest was provided more commonly in smoking interventions (88%) than wider psychology (39%) [19], social sciences (39%) [20], and biomedical science reports (65%) [21]. Seventeen percent of studies reported conflicts from private companies and 3% from tobacco companies. The comparatively high level of transparency markers observed here in smoking interventions compared to other fields is likely to reflect improved reporting following previous controversies in the field [25, 38, 39]. Funding and disclosure statements are now commonly mandated by journals related to smoking cessation [18, 26, 40].

### 4.3. Strengths and Limitations

A strength of this study is its use of double coding by two independent researchers of all reproducibility and transparency markers, enabling inter-rater reliability assessment. A limitation is that this study is based on a random sample of 100 evaluation reports of smoking behaviour change interventions, whereby assessments of reproducibility and transparency may not be generalizable to broader smoking interventions. Second, markers of reproducibility and transparency were dependent on what was described within evaluation reports. Direct requests to authors or additional wider searching of third-party registries such as Open Science Framework may have identified additional information indicating reproducibility. The absence of explicit statements on protocol, material, data, and analysis script availability may not necessarily signal that resources will not be shared by authors, but arguably does add an extra step for researchers to seek out this information. Third, this approach of assessing Open Science behaviours in reported research may omit more nuanced approaches to Open Science taken by journals or authors, which may make assessed figures lower than in actual practice.

### 4.4. Future Steps to Increase Reproducibility and Transparency of Smoking Interventions

Urgent initiatives are needed to address the low levels of reproducibility markers observed here in smoking intervention research, especially in the areas of open materials, data, analysis scripts, and replication attempts. As with any complex behaviour change, this transformation requires system change across bodies involved in smoking cessation research: researchers, research institutions, funding organisations, journals, and beyond [1, 7]. Interventions are needed to increase the capability, opportunity, and motivation of these bodies to facilitate behaviour change towards reproducible research in smoking interventions [28, 41]. For example, capability can be addressed by providing researcher training, equipping them with the skills needed to make their research open and reproducible, such as how to use the Open Science Framework, how to preprint servers, and how to make their analysis reproducible. Opportunity to engage in reproducible research in smoking interventions can be facilitated within institutions, facilitating discussions around open and reproducible working [42] and developing a culture around valuing progressive and open research behaviours [7].

Motivation to research reproducibly can be addressed by providing researcher incentives [7]. Open Science badges recognising open data, materials, and pre-registration have been adopted by journals as a simple, low-cost scheme to increase researcher motivation to engage in these reproducibility behaviours [43]. Open Science badges have been identified as the only evidence-based incentive program associated with increased data sharing [44]. However, adoption of Open Science badges in smoking cessation journals is currently low, indicating this as one important initiative currently missing in this field. Future research could compare this study's baseline assessment of reproducibility and transparency markers in smoking cessation intervention evaluation reports to assess changes in reporting and researcher behaviour.

## 5. Conclusions

Reproducibility markers of smoking behaviour change intervention evaluation reports were varied. Pre-registration of research plans and open access publication were common, whereas the provision of open data, materials, and analysis was rare and replication attempts were nonexistent. Transparency markers were common, with funding sources and conflicts of interest usually declared. Urgent initiatives are needed to improve reproducibility in open materials, data, analysis scripts, and replication attempts. Future research can compare this baseline assessment of reproducibility and transparency in the field of smoking interventions to assess changes.

## Figures and Tables

**Table 1:** Measured variables and operationalization.

Variables	Coder questions	Response options
Article characteristics		
Coder instructions: to identify journal impact factors use the Thomson Reuters Journal Citation Reports (https://library-guides.ucl.ac.uk/az.php?q=journal%20citation%20reports). For country, check the institutional affiliation of the corresponding author. If there are multiple corresponding authors, choose the first. If no corresponding author is identified, choose the first. If there are multiple affiliations for the selected author, choose the first.		
Journal impact factor 2018	What is the 2018 journal impact factor?	
Country	Which country is the corresponding author based in according to their affiliation?	[list countries]/unclear/other
*Smoking outcome behaviour*	*Already extracted as part of the Human Behaviour-Change Project*	*Smoking cessation/smoking reduction/second-hand smoking*
*Behaviour change techniques (BCTs) in all study groups*	*Already extracted as part of the Human Behaviour-Change Project*	*93 BCTs of the Behaviour Change Techniques Taxonomy v1* [30] *plus the addition of BCT 4.5 “advise to change behaviour”*
Reproducibility		
Preregistration		
Definitions: “preregistration” refers to the specification of important aspects of the study (typically hypotheses, methods, and/or analysis plan) prior to commencement of the study. Coder instructions: check specific sections in the paper where these files might be located, e.g., supplementary materials, appendices, author notes, methods, and results sections. Search for “registration”.		
Preregistration statement	Does the article state whether or not the study (or some aspect of the study) was preregistered?	Yes—the statement says that there was a preregistration/yes—the statement says that there was no preregistration/no—there is no preregistration statement/other^∗^
Preregistration method	Where does the article indicate the preregistration is located?	Open Science Framework/AsPredicted/ClinicalTrials.gov/AEA trial registry/EGAP registry/tegistered report/other^∗^
Preregistration accessible	Can you access and open the preregistration?	Yes/no/other^∗^
Preregistration content	What aspects of the study appear to be preregistered? (select all that apply)	Hypotheses Methods Analysis plan Other^∗^
Protocol sharing		
Definition: “protocol” refers to a document containing details about the study design, methods, and analysis plan. It may or may not be preregistered. Coder instructions: search the article for the phrase “protocol” and assess whether a link is provided to a protocol document.		
Protocol availability	Does the article link to an accessible protocol?	Yes/no/other^∗^
Protocol content	What aspects of the study appear to be included in the protocol? (select all that apply)	Hypotheses Methods Analysis plan Other^∗^
Data sharing		
Definitions: “data” refers to recorded information that supports the analyses reported in the article. A “data availability statement” can be as simple as a url link to a data file or as complex as a written explanation as to why data cannot be shared. Coder instructions: check the article for a data availability statement/link. They are often located in the “supplementary material,” “acknowledgements,” “author notes,” “methods,” or “results” sections. Search the article for the text “data availab” (to cover “data availability” and “data available”).		
Data availability statement	Does the article state whether or not data are available?	Yes—the statement says that the data (or some of the data) are available/yes—the statement says that the data are not available/no—there is no data availability statement/other^∗^
Data sharing method	How does the statement indicate the data are available?	Upon request from the authors/personal or institution website/an online, third-party repository (e.g., OSF and FigShare)/supplementary materials hosted by the journal/other^∗^
Data accessibility	Can you access, download, and open the data files?	Yes/no/other^∗^
Data documentation	Are the data files clearly documented?	Yes/no/other^∗^
Data content	Do the data files appear to contain all of the raw data necessary to reproduce the reported findings?	Yes/no/unclear/other^∗^
Material sharing		
Definitions: “material” refers to any study items that would be needed to repeat the study, such as stimuli, survey instruments, and computer code/software used for data collection, presentation stimuli, or running experiments.		
Material availability statement	Does the article state whether or not materials are available?	Yes—the statement says that the materials (or some of the materials) are available/yes—the statement says that the materials are not available/no—there is no materials availability statement/other^∗^
Material sharing method	According to the statement, how are the materials accessible?	Upon request from the authors/personal or institution website/an online, third-party repository (e.g., OSF and FigShare)/supplementary materials hosted by the journal/other^∗^
Material accessibility	Can you access, download, and open the material files?	Yes/no/other^∗^
Analysis script sharing		
Definition: “analysis scripts” refers to the specification of data preparation and analysis steps in the form of highly detailed step-by-step instructions for using point-and-click software, analysis code (e.g., R), or syntax (e.g., from SPSS). Coder instructions: check the article for an analysis script availability statement/link. They are often located in the “supplementary material,” “acknowledgements,” “author notes,” “methods,” or “results” sections. Search for the text “analysis script” and “analysis code”.		
Analysis script availability statement	Does the article state whether or not analysis scripts are available?	Yes—the statement says that the analysis scripts (or some of the analysis scripts) are available/yes—the statement says that the analysis scripts are not available/no—there is no analysis script availability statement
Analysis script sharing method	According to the statement, how are the analysis scripts accessible?	Upon request from the authors/personal or institution website/an online, third-party repository (e.g., OSF and FigShare)/supplementary materials hosted by the journal/other^∗^
Analysis script accessibility	Can you access, download, and open the analysis script files?	Yes/no/other^∗^
Replication		
Definitions: “replication” refers to the repetition of a previous study's methods in order to ascertain whether similar findings can be obtained. Coder instructions: search the abstract and introduction for the phrase “replicat” (to cover “replication,” “replicates,” etc.). Confirm the authors are using the phrase with the definition provided above.		
Replication statement	Does the article claim to report a replication study?	The article claims to report a replication study (or studies)/there is no clear statement that the article reports a replication study (or studies)/other^∗^
Open access		
Coder instructions: to establish the open access status of the article: Go to https://openaccessbutton.org/ and enter the article's doi (e.g., “10.1371/journal.pcbi.1004574”) if available (if not, enter the article title). If a link is provided, check that you can access the article at the link. If the article is accessible, answer “yes.” If the article is not accessible at the provided link, or no link is provided, answer “no.”		
Open access status	Is the article open access?	Yes—found via open access button/yes—found via other means/no—could not access article other than through paywall/other^∗^
Transparency		
Funding		
Coder instructions: funding is usually reported in a specific section, e.g., “author information” or “funding statement.” Search the article for the phrase “funding”. If you are unsure whether an organisation is a tobacco company, pharmaceutical company, other private company, or public organisation, Google the organisation name and code accordingly. If it is unclear to you whether the funding is private or public, choose the “other” option and enter “unclear”.		
Funding statement	Does the article include a statement indicating whether there were funding sources?	Yes—the statement says that there was funding from a tobacco company (e.g., Phillip Morris, British American Tobacco, China Tobacco, and Imperial Brands)/yes—funding from a pharmaceutical company (e.g., Pfizer and GSK)/yes—funding from another private company/yes—funding from a public organisation (e.g., National Institute of Health Research)/yes—the statement says that there was no funding provided/no—there is no funding statement/unclear/other^∗^
Conflict of interest		
Coder instructions: conflicts of interest are usually reported in a specific section, e.g., “author information” or “conflict of interest statement.” Search the article for the phrases “conflict of interest” and/or “competing interest”. If you are unsure whether an organisation is a tobacco company, pharmaceutical company, other private company, or public organisation, Google the organisation name and code accordingly. If it is unclear to you whether the funding is private or public, choose the “other” option and enter “unclear”.		
Conflict of interest statement	Does the article include a statement indicating whether there were any conflicts of interest?	Yes—the statement says that there was a conflict of interest from a tobacco company/yes—conflict of interest from a pharmaceutical company/yes—conflict of interest from another private company/yes—conflict of interest from a public organisation (e.g., National Institute of Health Research)/yes—the statement says that there is no conflict of interest/no—there is no conflict of interest statement/other^∗^

^∗^If a response marked with an asterisk is selected, the coder is asked to provide more detail in a free text response box. Note: identified measured variables have been adapted from a previous study assessing the transparency and reproducibility in psychological sciences [19].

## Data Availability

All data are provided on OSF: https://osf.io/5rwsq/.

## Appendix

### Updates to Preregistered Protocol

During the course of this study and peer review, we made minor adjustments to the preregistered protocol as follows:

1. We revised the remit of “smoking cessation” to instead refer to “smoking behaviour change” more broadly. This allowed inclusion of cessation, reduction, and second-hand smoke intervention reports included within the Human Behaviour-Change Project knowledge system
2. Within the article characteristics measured variables, we added “smoking cessation behaviour” to identify whether each report addressed smoking cessation, reduction, or second-hand smoke specifically
3. Within the article characteristics measured variables, we added “behaviour change techniques” to specify the intervention content identified within each report. Behaviour change techniques were already coded within the parallel Human Behaviour-Change Project: working to synthesis published evidence in behaviour change, beginning with smoking intervention evaluations
